# Three-Dimensional Traction Force Microscopy: A New Tool for Quantifying Cell-Matrix Interactions

**DOI:** 10.1371/journal.pone.0017833

**Published:** 2011-03-29

**Authors:** Christian Franck, Stacey A. Maskarinec, David A. Tirrell, Guruswami Ravichandran

**Affiliations:** 1 School of Engineering, Brown University, Providence, Rhode Island, United States of America; 2 Division of Chemistry and Chemical Engineering, California Institute of Technology, Pasadena, California, United States of America; 3 Division of Engineering and Applied Science, California Institute of Technology, Pasadena, California, United States of America; Clarkson University, United States of America

## Abstract

The interactions between biochemical processes and mechanical signaling play important roles during various cellular processes such as wound healing, embryogenesis, metastasis, and cell migration. While traditional traction force measurements have provided quantitative information about cell matrix interactions in two dimensions, recent studies have shown significant differences in the behavior and morphology of cells when placed in three-dimensional environments. Hence new quantitative experimental techniques are needed to accurately determine cell traction forces in three dimensions. Recently, two approaches both based on laser scanning confocal microscopy have emerged to address this need. This study highlights the details, implementation and advantages of such a three-dimensional imaging methodology with the capability to compute cellular traction forces dynamically during cell migration and locomotion. An application of this newly developed three-dimensional traction force microscopy (3D TFM) technique to single cell migration studies of 3T3 fibroblasts is presented to show that this methodology offers a new quantitative vantage point to investigate the three-dimensional nature of cell-ECM interactions.

## Introduction

The exchange of physical forces in cell-cell and cell-matrix interactions plays a significant role in regulating a variety of physiological and pathological processes including wound healing, angiogenesis, metastasis and embryogenesis [1]–[3]. For example, benign to malignant phenotype transformation has been shown to be strongly influenced by the microstructure and mechanical signature of the surrounding extracellular matrix (ECM), particularly in three-dimensional environments [4]–[6]. Hence, quantification and understanding of the nature of cell-ECM interactions and regulation within three-dimensional environments become important for the development of new biomaterials and clinical diagnostics. Within the last few decades, studies have begun to quantify traction forces that are developed by migrating cells through a variety of techniques. For example, in 1980 Harris et al. demonstrated that cellular forces could be visualized by tracking the wrinkling formation of thin elastic silicone rubber substrates due to applied cell stresses [7]. However, since wrinkling is an intrinsically nonlinear and unstable process, the quantitative characterization using this technique is difficult. In 1995 Oliver et al. and Dembo et al. developed a quantitative technique called traction force microscopy (TFM) to study fibroblast migration on two-dimensional substrate surfaces [8]–[10]. While other experimental techniques, such as micropillars and embedded force sensors have made significant contributions in quantifying cell-matrix interactions [2], [11], traction force microscopy remains the most widely used methods for measuring cellular traction forces [12]–[16].

Traction force microscopy utilizes optical phase and wide-field microscopy to track substrate surface displacements due to cellular traction forces through the spatial correlation of fluorescent particles embedded in the substrate. Polyacrylamide gels are among the most commonly used substrate materials in studying cell force responses due to their mechanical tunability, optical translucency, and elastic material behavior [17]. By controlling the mole fraction of added crosslinker N, N-methylene-bis-acrylamide (BIS), the Young's modulus of each polyacrylamide gel can be controlled, with typical moduli ranging from 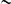
1–30 kPa, in the range of physiologically relevant moduli [13], [15], [16], [18].

To record cell surface deformations, cells are initially seeded on the substrate material and allowed to spread or migrate. After some time, a first image is captured optically, where typically both the cell and tracker particles are recorded simultaneously. Then, cells are detached from the surface through trypsinization or similar treatment. A second image is captured (without moving the microscope's objective) to serve as the undeformed or reference configuration. Cell-induced substrate displacements are then determined from the two images by using either a single particle tracking or a digital image correlation algorithm. The resulting gel displacements are converted into traction forces using the inverse Boussinesq formulation, where the Boussinesq theory describes the displacement equilibrium solutions inside a semi-infinite elastic half-space with applied forces at its free boundary [19]. However, since the Boussinesq formulation needs to be utilized inversely to compute cell traction forces, it has the complication that the solution is no longer unique and the computation itself can become expensive. Hence, additional regularization and iteration algorithms are needed to provide a stable solution [13], [15], [20]. Although the Boussinesq solution provides an approach to determine surface traction forces from measured displacements directly, it is also dependent on the assumption of a semi-infinite elastic half-space or an elastic substrate of infinite thickness. Determining when a substrate can be treated as infinitely thick is difficult in the absence of any direct information about the extent of deformation in the third spatial dimension (i.e., the thickness direction). It has been shown that the Boussinesq solution underestimates the computed traction force when cells are seeded on gels ranging in thickness from 5–60 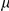
m, and that finite height corrections are necessary [21], [22]. The Boussinesq solution also requires that the displacement data is indeed recorded at the free surface, which can be difficult to determine without depth information.

While previously published traction force microscopy studies have contributed a great deal to our understanding of cell behavior and local cell-ECM interactions, they remain inherently restricted to two dimensions. Current cell motility and mechanotransduction models are based on experimental findings from two-dimensional studies [23], [24]. However, many physiological processes are three-dimensional in nature and recent studies have shown morphological differences in cells cultured on two-dimensional substrates versus three-dimensional matrices. ECM interactions and migration behavior also differ in two and three dimensions [25]–[27].

This study presents a three-dimensional traction force microscopy (3D TFM) technique capable of measuring cellular deformations in three dimensions with submicron accuracy. While this technique has been used previously to measure deformation fields within soft biomaterials and around migrating fibroblast cells [28], [29], the details of this method have not been fully described. Here we discuss how 3D TFM can be used to extract three-dimensional displacement and force information for motile cells. We also present, for the first time, direct three-dimensional experimental evidence of a cellular detachment peeling mechanism during locomotion. In this approach, cell-induced three-dimensional displacement and strain fields are experimentally determined by tracking the motion of submicron fluorescent markers embedded in hydrogels such as polyacrylamide using laser scanning confocal microscopy (LSCM) and digital volume correlation (DVC) [28], [29].

A similar methodology has been demonstrated by Hur et al. by combining LSCM with finite element modeling (FEM) to compute the resulting cell traction fields [30]. Although this approach requires FEM calculations to determine the full three-dimensional traction fields, it provides high temporal resolution by scanning only the surface displacements directly underneath the cell. Hence it provides a robust technique for investigating planar and easily meshable substrate geometries. However, its application to fully encapsulated cell-matrix systems is more challenging due to the requirements of complex mesh generations for encapsulated cells and non-uniform cell boundaries in three dimensions. Recently, Gjorevski et al. used a similar combination of confocal microscopy, TFM and FEM to demonstrate that the pattern of branching morphogenesis of 3D epithelial tissues occurs at regions of elevated mechanical stress and that the extent of branching in this tissue model correlates with the magnitude of stress at branching sites [31]. In another recent investigation Legant and coworkers measured three-dimensional traction forces exerted by fibroblasts encapsulated in bioactive hydrogels and discovered that cells in a 3D matrix largely impart shear stresses which increase as a function of increasing distance from the center of mass of the cells, with regions of the highest stress localized near the tips of long projections. The calculation of traction stresses in this study was accomplished by generating a finite element mesh surrounding a cell in a hydrogel using confocal images. The authors then implemented a discretized Greens function to the finite element mesh and solved the finite element equations to calculate the bead displacement by comparing images collected before and after cell lysis [32]. While these recent investigations have provided us with a better understanding of the importance of 3D traction forces during cellular events, their mathematical formulations typically require additional resources such as FEM packages [30]–[32] or solutions to inverse problems, which tend to introduce significant noise [32].

Here we describe straightforward and accurate calculations of cellular displacements and tractions via the 3D TFM technique, without the need to invoke complex mathematical frameworks, for both planar and fully three-dimensional systems. The 3D TFM methodology is utilized to investigate the dynamic evolution of traction forces during migration of 3T3 fibroblasts on polyacrylamide substrates.

## Results

Full field displacement measurements were carried out using the LSCM-DVC technique applied to migrating 3T3 fibroblast cells on polyacrylamide gels with a Young's modulus of E 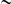
9.64 kPa. Although the Young's modulus of the polyacrylamide gels was not varied in this study, further comparison of 2D versus 3D cell behavior can be found elsewhere [25], [29], [33]. Confocal volume stacks were recorded at 35 minute time increments to capture the locomotion of individual cells over several hours. The results depict dynamic cell-ECM interactions of the same cell over several hours without chemical intervention, such as trypsinization to detach the cells from the substrate surface.

Each cell was visualized simultaneously with the displacement of the fluorescent particles inside the polyacrylamide gels using two separate photodetectors. This procedure allowed correlation of the position of the cell (determined by GFP-actin) with the substrate displacement field. However, due to the finite life-time and degradation of the GFP, parts of the cell were occasionally not visible at locations where substantial deformations were observed.

**Figure 1:**
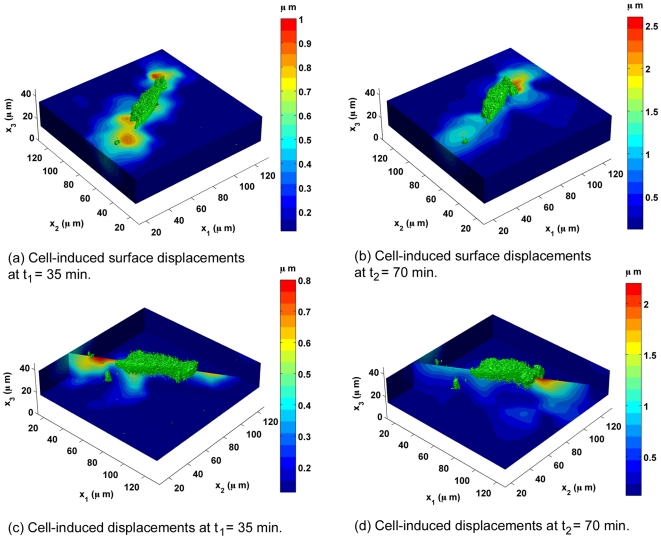
3D Displacement Contours of Migrating Fibroblast. Surface contour ((a)–(b)) and depth contour ((c)–(d)) plots of the magnitude of the three-dimensional displacement vector (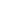 **u** 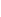) at two time points 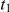 and 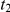 (separated by a 35 min interval) during cell migration. The color bar represents the magnitude of the total three-dimensional displacement vector (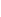 **u** 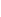) in 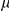
m, and the cell (green) is superimposed on the three-dimensional contour plots to show its position with respect to the deformation field. The two edges in (c) and (d) are included to show that there are negligible displacements detected from neighboring cells (contours are dark blue).

Figure 1 shows the time evolution of the three-dimensional displacement field beneath a single motile cell on the polyacrylamide substrate over a time span of 70 min. The color contour plots display the magnitude of the three-dimensional displacement vector in 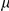
m. The confocal cell image shown by the intensity distribution of GFP-actin is superimposed on the displacements field to correlate the location of the cell with the detected displacement fields. Figure 1 shows a highly polarized cell with displacement concentrated at the leading and trailing edges of the cell. The longer dimension of the cell in all of the plots is approximately 80–100 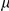
m. The direction of cell locomotion is from the lower left to the upper right, with changes in direction every two to four time frames and an average cell speed of 8 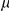
m/hr as determined by tracking the nucleus of the cell.

Figures 1(a) and 1(b) display the distribution of the magnitude of the three-dimensional displacement vector on the surface plane directly underneath the cell at two different time increments (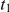 and 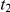) with a 35 min time interval between recordings. Figs. 1(c) and 1(d) display the magnitude of the three-dimensional displacement vector through the thickness of the polyacrylamide substrate at the same time points. The displacement contour slices highlight the dynamic interaction of the cell with its substrate characterized by changes in magnitudes and locations of the observed displacements, both along the surface plane (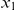
−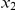) and through the substrate thickness (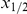
−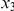). Since the three-dimensional full-field displacements are determined as shown in Fig. 1, the complete three-dimensional strain and stress tensors can be calculated without any *a priori* assumptions regarding the stress state (e.g., plane stress) and geometry (e.g., infinite substrate thickness). This removes several conditions needed to determine traction forces from two-dimensional data sets, such as an infinite substrate requirement or the inverse calculations required within the framework of the Boussinesq theory [13], [15], [20]. Here, since the three-dimensional stress state is known, the calculations of cell traction forces is straightforward using Eq. 13, and since all traction calculations are performed in a forward manner stabilizing schemes are not needed as in the case for typical inverse problems.

**Figure 2:**
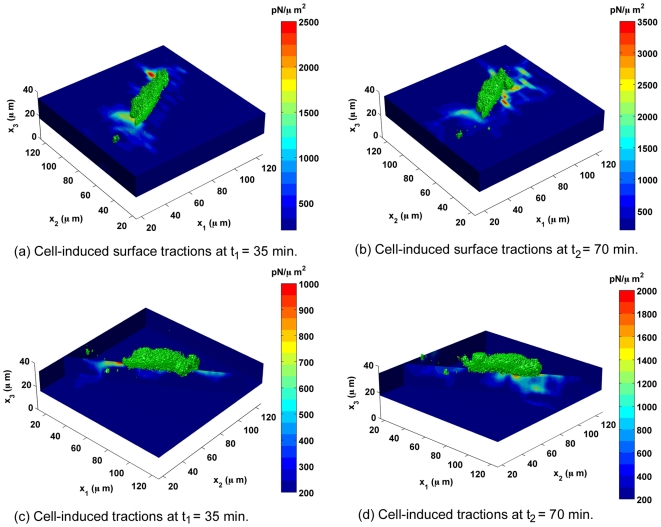
3D Traction Contours of Migrating Fibroblast. Surface contour ((a)–(b)) and depth contour ((c)–(d)) plots of the magnitude of the three-dimensional traction force vector, corresponding to the displacement fields presented in Fig 1. The color bar represents the magnitude of the total three-dimensional traction force vector (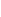 **T** 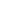) in pN/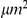, and the cell (green) is superimposed on the three-dimensional contour plots to show its position with respect to the traction field. The two edges in (c) and (d) are included to show that there are negligible traction forces detected from neighboring cells (contours are dark blue).

Figure 2 shows the traction fields calculated from the displacement data in Fig. 1. The color contours display the magnitude of the three-dimensional traction force vector. At both time points, 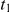 and 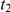, the data show highly localized traction fields with a peak magnitude directly underneath the cell near the cell edge, most likely corresponding to local force transmission at focal adhesions at both the trailing and leading edges of the cell.

Figures 2(c) and 2(d) highlight the depth distribution of cellular traction forces underneath the cell. The dynamic mechanical interaction of the cell and its substrate is highlighted in both time frames by changes in the location and magnitude of the traction forces. As in Fig. 1, the outline of the cell is shown in green and superimposed on the traction field data.

**Figure 3:**
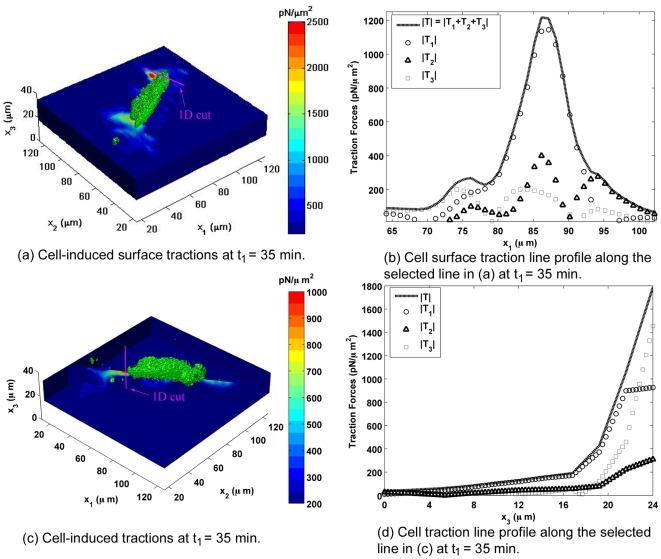
Distribution of 3D Cellular Traction Forces on Polyacrylamide Gels. Cell-induced surface traction forces contour plot ((a)) at 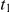 = 35 min during migration. The color bar represents the magnitude of the three-dimensional surface traction force vector (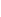 **T** 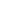) in 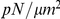. The pink line depicts the location of the generated one-dimensional plot shown in (b). The location was chosen to show the line profile across a localized force concentration (see Figure S1 for expanded view); (b) illustrates the distribution of the magnitude of the total traction force vector and its in-plane (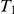, 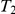) and normal (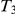) components along the selected line-cut highlighted in (a); (c) and (d) display the cell-induced traction force contour and line plot profiles as functions of depth (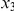) through the thickness of the gel (see Figure S2 for expanded view); (c) shows the same traction force contours along the long axis of the cell where the color bar represents the magnitude of the three-dimensional traction force vector (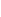 **T** 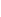) in 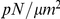; (d) illustrates the distribution of the magnitude of the total traction force vector (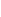 **T** 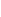) and its in-plane (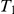, 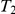) and normal (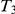) components in the thickness (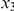) direction.

**Figure 4:**
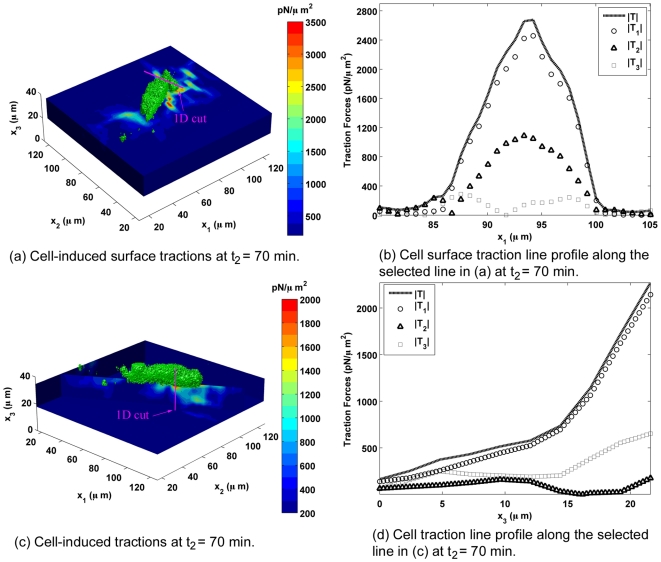
Distribution of 3D Cellular Tractions on PA Gels. Cell-induced surface traction forces contour plot ((a)) for the next time point (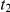 = 70 min) during migration. The color bar represents the magnitude of the three-dimensional surface traction force vector (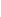 **T** 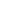) in 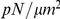. The pink line depicts the location of the generated one-dimensional plot shown in (b). The location was chosen to show the line profile across a localized force concentration; (b) illustrates the distribution of the magnitude of the total traction force vector (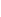 **T** 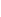) and its in-plane (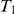, 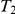) and normal (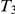) components along the selected line-cut highlighted in (a); (c) and (d) display the cell-induced traction force contour and line plot profiles as a function of depth (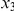) through the thickness of the gel; (c) shows the same traction force contours along the long axis of the cell where the color bar represents the magnitude of the three-dimensional traction forces in 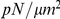; (d) illustrates the distribution of the magnitude of the total traction force vector and its in-plane (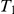, 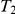) and normal (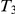) components in the thickness (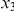) direction.

Figures 3 and 4 show line profiles of the traction forces for time points 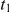 and 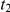, respectively. Fig. 3(b) displays a one-dimensional profile of the magnitude (absolute value) of each surface traction force component (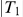, 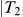, 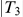) including the magnitude of the total three-dimensional traction force vector 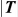 along the plotted line in Fig. 3(a). The line profile is drawn across an area of concentrated traction forces at the cell's leading edge likely corresponding to a focal adhesion site (see Figure S1 for a zoomed, more detailed view). The magnitude of the total traction force vector follows a Gaussian profile with a peak value of 1.22 nN/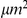 over an area of 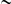
1 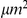, which is similar to previous measurements of traction magnitudes and focal adhesion size [14], [15], [34]. For this location and time increment, the in-plane shear tractions 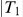 and 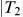 dominate the out-of-plane tractions with magnitudes 2–3 times larger than 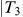.

Figure 3(d) displays the decay in the magnitude of the three-dimensional traction force vector 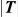 and its components (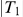, 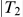, 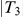) through the thickness of the substrate at the trailing edge of the fibroblast (see Figure S2 for a zoomed, more detailed view). On the surface, the ratio of the out-of-plane traction force component 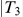 to both the in-plane components 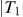 and 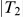 is 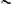
1.6 and 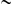
5, respectively, and thus 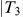 is the major contributing traction force component. The traction profile follows a rapid decay characterized by a 90% drop in the magnitude within the first 7 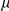
m below the substrate surface.

Figures 4(b) and 4(d) present traction force line profile plots for the next time point 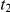. Figure 4(b) shows the distribution of the magnitude of the three-dimensional traction force vector and its component along the highlighted line in Fig. 4(a). Again, the traction force 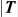 curve exhibits a Gaussian shape with a peak value of 2.68 nN/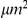 over an area of 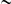
1 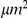. Figure 4(b) reveals a similar but broader traction force profile when compared to Fig. 3(b). Similar to Fig. 3(b), the in-plane (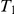, 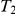) shear traction forces are the major constituents of the overall traction force vector with maximum values of 2.5 nN/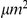 and 1.1 nN/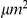 for 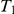 and 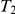, respectively. Figure 4(d) depicts the distribution of the magnitude of the three-dimensional traction force vector and its components through the substrate thickness along the highlighted line in Fig. 4(c). The magnitude of the traction forces decay in similar fashion as shown in Fig. 3(d), however, the progression is more gradual and is dominated by in-plane shear stresses (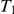).

**Figure 5:**
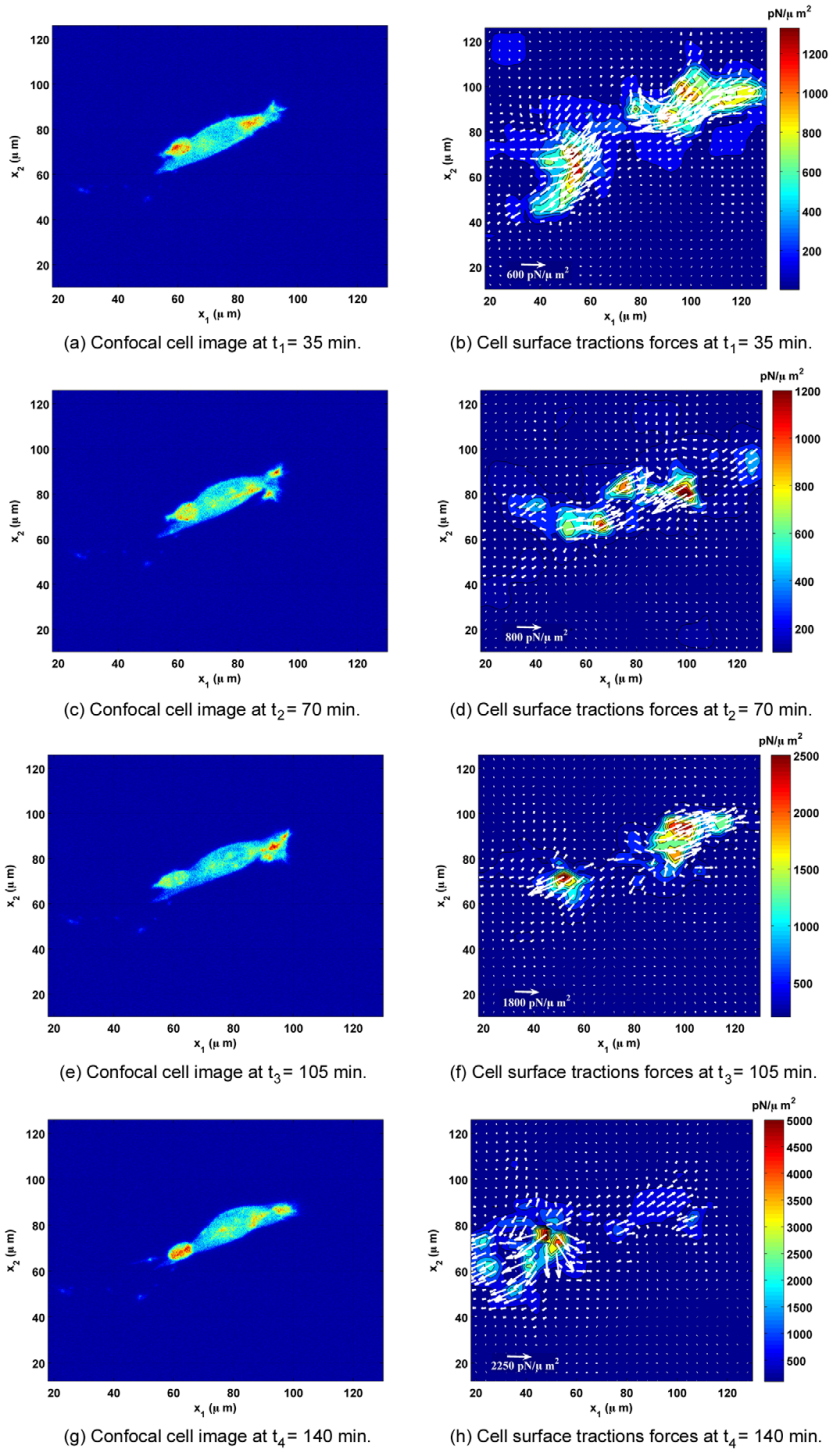
Time Evolution of 3D Displacements and Tractions of a Migrating Fibroblast. Time evolution of a successive series of laser scanning confocal cell images (left column) and traction force contours (right column) on the surface during cell migration for a single cell. The cell images on the left represent two-dimensional projections of the confocal volumetric data set showing GFP-actin. The cell-applied surface traction force contours display the magnitude of the three-dimensional traction force vector (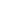 **T** 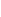) in 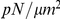. The white arrows represent the in-plane traction force components (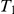 and 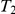) only.

Figure 5 shows a longer time series for the same cell as shown in Figs. 1, 2, 3, 4, displaying a confocal cell image on the left during each 35 min time increment and its corresponding surface traction force field directly underneath the cell on the right. The color contour plots show the magnitude of the three-dimensional traction force vector (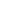 **T** 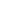), while the white arrows display the in-plane components (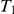, 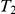) only. The general direction of cell locomotion is from left to right with a translocation of the nucleus ranging from 0.9–4.3 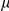
m during each time increment. Since the confocal microscope records depth information (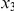), precise selection of the plane directly underneath the cell is possible, thereby eliminating any ambiguity in depth location of the presented traction fields. Comparing Figs. 5(a)–5(h) the cell appears to actively probe its environment during locomotion characterized by either localized contraction (5(b) and 5(d)) or extension (5(f) and 5(h)). In each frame localized maxima in surface tractions are clearly visible with a typical spatial size on the order of 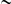
1 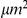, which is consistent with previous observations of focal adhesion sites [14].

## Discussion

As illustrated in Figs. 1, 2, 3, 4, 5, fibroblast-matrix interactions are orchestrated in a dynamic and three-dimensional fashion during locomotion on polyacrylamide substrates. While previous studies have given significant insight into cell-matrix interactions in primarily two dimensions by reporting traction forces and total detachment forces for cells, this study provides a more detailed picture of cell-substrate interactions in three dimensions during different stages of locomotion. Comparison of the traction forces and displacements with previously published two-dimensional data sets, and the recently published three-dimensional study on bovine aortic endothelial cells shows similar spatial distribution, localization and magnitudes of cell-applied traction forces and displacements [13]–[16], [30].

As shown earlier, cell-induced deformations can extend through 50% of the substrate thickness or up to 25% of cell's body length (long axis). Unless experiments are designed with such considerations in mind, it is likely that cells will respond to an effective modulus that is a function of film thickness, rather than to the modulus of the gel layer alone [35].

**Figure 6:**
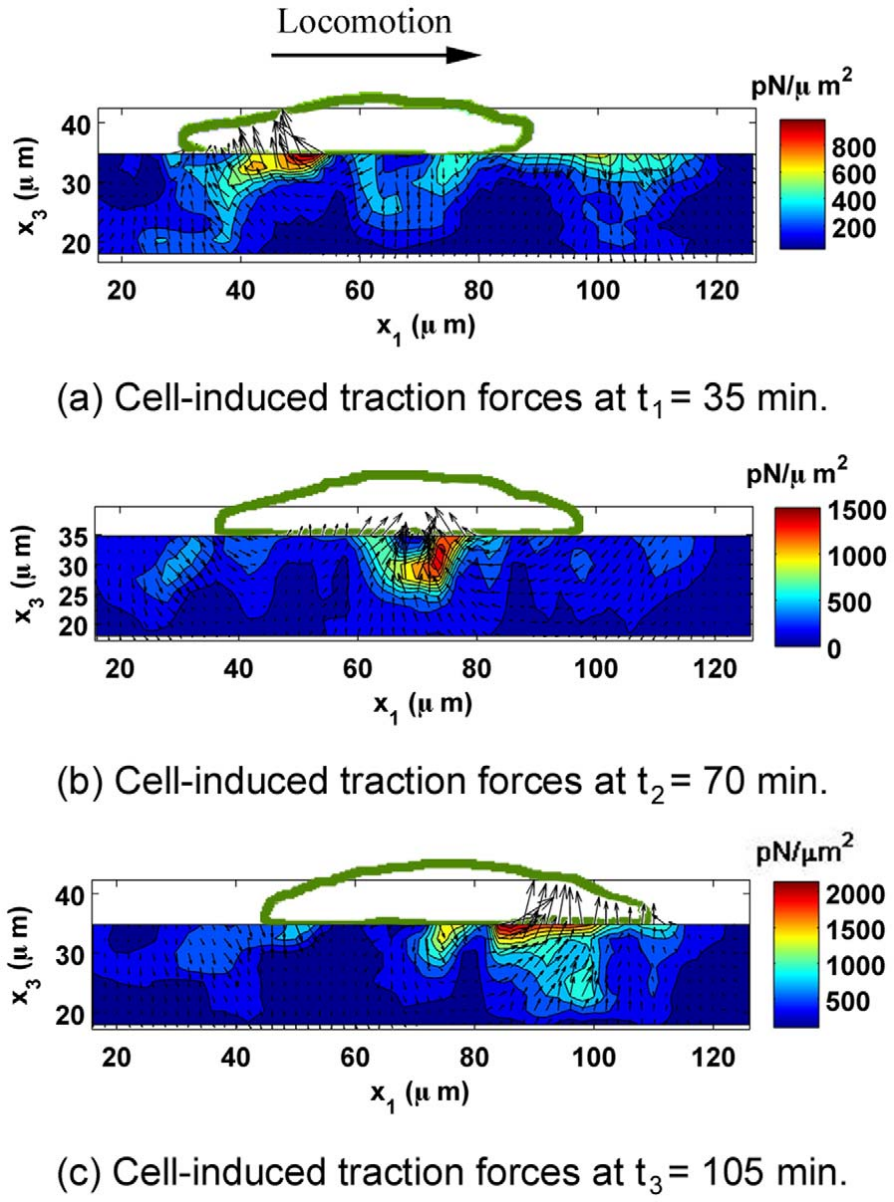
Time Evolution of 3D Traction Forces through the Gel Thickness. Time evolution of cell-induced traction forces as a function of depth (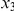) over 70 min along an arbitrary slice below the cell's long axis. The contour plots show the magnitude of the three-dimensional traction force vector (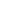 **T** 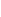) for a single locomoting 3T3 fibroblast in 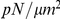. The black arrows represent the in-plane traction forces (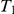) and normal traction forces (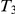), where the magnitude of the longest arrow during each time increment is equal to the maximum value depicted by the color bar in 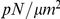. The time increment between successive frames is 35 min. The outline of the cell as recorded by confocal imaging is superimposed in green, and the direction of cell migration is from left to right.

**Figure 7:**
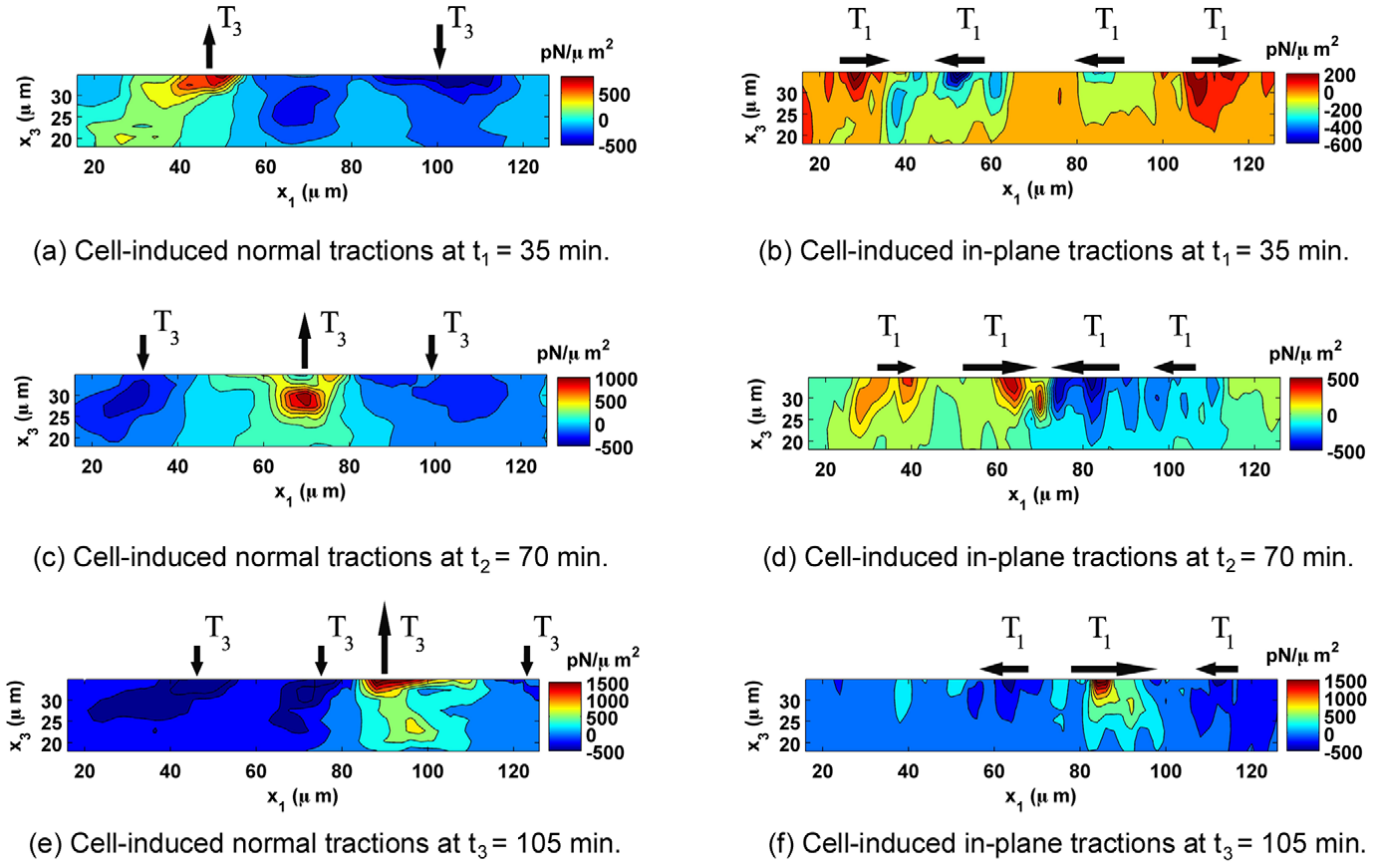
Decomposition of the Local 3D Cell Tractions During Locomotion. Time evolution of cell-induced traction forces 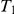 (in-plane) and 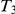 (normal) as a function of depth (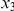) over 70 min along an arbitrary slice below the cell's long axis as shown in Fig. 6. The contour plots show the magnitude of the shear traction force components (left column: 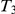; right column: 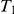). The color bar displays all values in 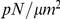. The black arrows on the top of each plot give the general direction of cell-induced traction forces. The time increment between successive frames is 35 min. The direction of cell migration is from left to right.

This study also shows that cells can actively modulate in-plane (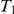, 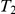) and out-of-plane (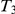) matrix stresses while exploring their local environment (e.g., Figs. 3(d) and 4(d)). These results show that the normal traction force component, (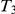), is equally important in cell-matrix interactions during locomotion as the previously measured in-plane (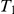, 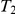) traction force components (observed for n = 32 samples). Figures 6 and 7 further illustrate this point by displaying the time evolution of the distribution of cell-generated traction forces through an arbitrarily chosen plane directly underneath the cell and normal to the gel surface (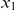
−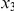). The color contour displays the magnitude of the three-dimensional traction force vector, while the in-plane vectors display the in-plane and normal components (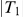, 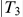). Figure 6 highlights the dynamic three-dimensional push-pull behavior of a fibroblast during locomotion from left to right. While the cell's leading edge (left) applies a downward force on the substrate, the trailing edge (right) applies an upward force, resulting in a three-dimensional peeling or rolling motion during movement from left to right. The cell outline captured during confocal imaging is superimposed onto the contour plots to show the location of the cell during each push-pull stage. Examining the magnitude of each of the 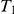 and 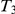 traction force components in Fig. 7 elucidates this mechanism in more detail. Figure 7 presents the same time evolution as shown in Fig. 6, however, now displaying the individual in-plane (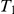) and normal (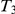) components as color contour plots. The black arrows are added to visualize the location and the relative magnitude of each local traction concentration more easily. The in-plane (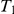) traction forces seem to alternate between local contraction and extension close to force equilibrium, while the normal (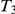) traction forces show a net moment around the center of the cell body in Figs. 7(a) and 7(e). This suggests that the cell is utilizing a more complex migration mechanism than previously suggested by two-dimensional data [23], [24], incorporating rotations along with planar contractions and extensions. Furthermore, notable tractions are observed underneath the cell nucleus in Figure 6b (Please see supplemental information Figure S3 for an expanded view). Although the nature of these forces is not further explored in this study, other reports have suggested that mechanical coupling of the nuclear envelope and the cytoskeleton through nuclear adhesion complexes may play a role in regulating nuclear shape, gene expression, and other downstream cellular processes [36].

Although Fig. 6 depicts discrete time events separated by 35 min intervals, it presents a new vantage from which to investigate the mechanism of cell detachment and to determine whether the process is consistent with previous models of the peeling of focal adhesion clusters [37], [38]. The 3D TFM technique provides a new perspective on the cell's complex migration machinery and should lead to the development of new three-dimensional cell motility models.

The 3D TFM method introduced here shows the viability of capturing cell-generated deformations in all three spatial dimensions with submicron accuracy. This technique enables the direct determination of three-dimensional displacement fields and thus direct calculations of traction forces using principles of mechanics without the need to resort to particular mathematical frameworks (e.g., Boussinesq theory) or FEM-based analysis. The study highlights the highly three-dimensional deformation fields around migrating 3T3 fibroblasts and shows the local dynamic force modulation of these cells at different times. It also presents experimental evidence for a three-dimensional peeling or rolling motion during cell locomotion characterized by changes in the orientation and magnitude of the cell-induced three-dimensional traction forces. While this study focused on single cell matrix interactions, future studies might envision quantifying cell-substrate interactions for multiple cells, cell sheets or cell clusters. Since the technique can accurately resolve traction forces as a function of time in three dimensions, it should create new avenues for quantifying cell-matrix interactions in three-dimensional tissues (e.g., encapsulated cells) and biomaterials and provide a tool for studying the intrinsic coupling of mechanical and biochemical interactions outside and inside cells in all three dimensions.

## Materials and Methods

### Three-dimensional Traction Force Microscopy (3D TFM)

**Figure 8:**
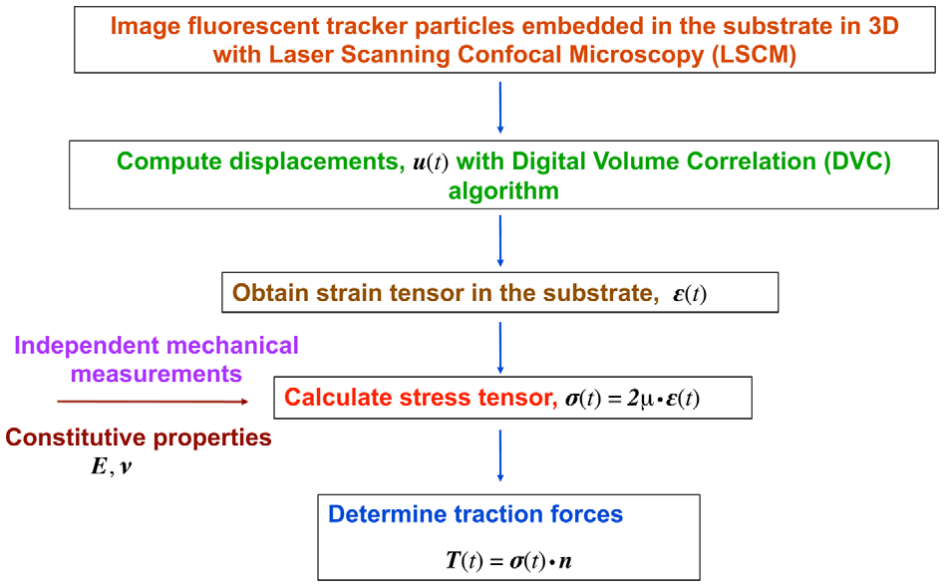
3D Traction Force Microscopy Overview. Schematic overview of the three-dimensional traction force microscopy technique, illustrating the methodology to compute the full-field tractions of migrating cells in all three spatial dimensions.

This section provides a general overview of the newly developed three-dimensional traction force microscopy technique. This method has the capability of determining the three-dimensional cell-induced full-field displacements and traction forces inside transparent biomaterials, such as polyacrylamide and collagen gels. Figure 8 provides a schematic overview of this technique showing how cell-induced traction forces are computed in all three spatial dimensions.

First, submicron-sized fluorescent particles are embedded within the transparent biomaterial of interest and three-dimensional time-lapsed volumetric images are recorded using laser scanning confocal microscopy (LSCM). Next, cell-induced material deformations (i.e., displacements) are calculated using a recently developed digital volume correlation (DVC) algorithm that tracks the center displacement of individual spatial subsets of the recorded LSCM images in all three dimensions [28]. Once the full-field displacements are obtained the three-dimensional strain tensor (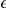) is calculated using a previously described displacement-gradient method [28]. To calculate the cell-induced three-dimensional full-field stresses or the stress tensor (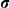), the material properties (e.g., Young's modulus and Poisson's ratio) or the material's constitutive behavior need to be determined through independent mechanical testing. Finally, cell-applied traction forces can be directly calculated along any plane (represented by its normal vector (**n**)),within the material as well as on its surface, by simple matrix multiplication of the normal with the material stress tensor. As the schematic illustrates, this method does not rely on any mathematical frameworks or stress-state assumptions, such as the Boussinesq theory or FEM analysis. Because all material displacements can be calculated from three-dimensional LSCM images, the tractions can be calculated in a forward way based solely on mechanics principles as described in detail in the following sections. This obviates the need for any inverse formulation and stabilization schemes that are generally necessary for 2D TFM calculations.

**Figure 9:**
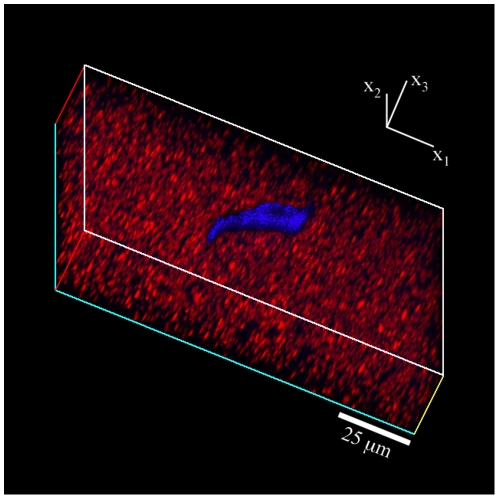
3D LSCM Cell Image. 3D LSCM image of a polyacrylamide gel including embedded submicron tracker particles (red) and a fibroblast cell on the surface (blue). The total stack dimensions are 128×128×30 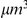. The rendered cell image has been digitally enhanced in brightness and contrast and superimposed onto the confocal stack to show a clearer image.

Figure 9 shows an example of a three-dimensional volumetric image recorded using LSCM. The submicron particles are shown in red and the cell outline is superimposed in blue. Figure 9 was rendered from an actual confocal scan but the colors have been digitally enhanced to provide better contrast between the cell outline and the embedded tracker particles. The details of the three-dimensional traction force microscopy method introduced here are presented in the subsequent sections.

### Preparation of activated coverslips

Glass coverslips (Gold-Seal coverslip No. 0, Electron Microscopy Sciences) were chemically modified to allow for covalent attachment of polyacrylamide sheets using a previously established protocol [17], [34]. Briefly, coverslips were rinsed with ethanol and then placed in a dish containing a solution of 0.5% (v/v) 3-aminopropyltrimethoxysilane (Gelest) in ethanol for 5 minutes. The coverslips were removed from the dish and rinsed thoroughly with ethanol before being immediately immersed in a solution of 0.5% glutaraldehyde (Polysciences, Inc) and water for 30 minutes. Activated coverslips were rinsed thoroughly with deionized water and left to dry for several hours at 60

C. Treated coverslips were then covered and stored for up to one week after preparation.

### Preparation of polyacrylamide films

Polyacrylamide films were generated and fused to functionalized coverslips using a previously adapted protocol [15], [34]. Solutions of polyacrylamide (Bio-Rad, 40% w/v) and N, N-methylene-bis-acrylamide (BIS, Bio-Rad, 2.5% w/v) were mixed with distilled water to obtain final volume fractions of 10% acrylamide and 0.015% BIS, respectively. Next, fluorescent micro-particles (0.5 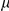
m in diameter, carboxylate-modified, Molecular Probes) in a 2% (w/v) suspension were vortexed for 10–15 seconds and added to the polyacrylamide solution in a volume ratio of 9∶100. Crosslinking was initiated through the addition of ammonium persulfate (Sigma) and N,N,N,N-tetramethylethylenediamine (Invitrogen). The samples were vortexed for 10 seconds, and 5–7 microliters of the acrylamide solution was pipetted on the surface of a precleaned microscope slide (No. 1, 22 mm×50 mm, VWR) yielding a typical gel thickness around 30 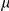
m. To generate thicker films, 20–40 microliters of the solution were used. The thickness of each sample was measured by vertical slicing of the acquired volumetric confocal images, and calculating the distance from the top to the bottom layer of fluorescent particles. The activated surface of the coverslip was then placed on top of the acrylamide droplet, causing the solution to flatten under the weight of the coverslip. Polymerization was allowed to proceed at room temperature for 5 minutes, and the substrates were placed in a 60 mm Petri dish (VWR) containing distilled water for 10–30 minutes. The bottom coverslip was peeled off using a pair of tweezers, leaving the polyacrylamide gel bonded to the activated coverslip. The polyacrylamide gel was thoroughly rinsed with water and hydrated in a 60 mm Petri dish.

### Functionalization of polyacrylamide substrates with fibronectin (FN)

In order to promote cell attachment to polyacrylamide films, fibronectin was conjugated to the gel surface using the heterobifunctional crosslinker, sulfo-SANPAH (Pierce Chemicals). Adopting a previously outlined procedure [15], polyacrylamide gel samples were briefly dried in air to remove any excess water before 200 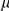
l of sulfo-SANPAH (1.0 mg/ml) were deposited on the surface of the film. The sample was exposed to unfiltered UV light from a high-pressure mercury lamp (Oriel Q 100W at 5 A, 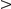
10 min warm up time) at a distance of 2.5 cm for 7.5 minutes. The darkened sulfo-SANPAH solution was removed from the surface of the gel and replaced with another 200 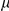
l aliquot solution of sulfo-SANPAH and irradiated for another 7.5 minutes for a total of 15 minutes of UV exposure. The samples were rinsed vigorously with water for 5 minutes, and adhered to the bottom of 60 mm Petri dishes (Becton Dickinson) by applying a thin layer of vacuum grease (Dow Corning) around the perimeter of the unmodified side of the coverslip. The samples were rinsed twice with phosphate buffered saline (pH 7.4), covered with a solution of fibronectin (FN, 0.2 mg/ml, Millipore) and left undisturbed overnight at 4

C. Following overnight incubation, the substrates were rinsed three times with PBS. The typical thickness of the fibronectin layer was approximately 1 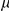
m as determined by LSCM.

### Cell culture

Prior to depositing cells, FN-modified gel samples were equilibrated in growth medium at 37

C for 15 minutes. Swiss 3T3 fibroblasts transfected with a GFP-actin vector were cultured in Dulbecco's Modified Eagle Medium (DMEM, Invitrogen) supplemented with 10% fetal bovine serum (Invitrogen), 50 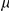
g/ml streptomycin (Invitrogen), and 50 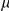
g/ml penicillin (Invitrogen). For all experiments, cells were first treated with Mitotracker Deep Red (Invitrogen) for 45 minutes before passaging with trypsin. Mitotracker dyes accumulate in actively respiring mitochondria providing a second method in addition to the GFP-actin vector for tracking the location of cells on the material as well as demonstrating cell viability. Cells were plated at concentration of 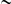
40,000 cells/coverslip, and incubated on samples for 8–12 hours before imaging. Cells were plated at a low density in order to allow for isolated cells to be imaged. Cells were considered isolated if no other cells could be visualized by confocal microscopy within 80–100 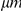 of the selected cell. Imaging of isolated cells ensures that nearby cells do not contribute to the measured displacements.

### Mechanical characterization of polyacrylamide substrates

The mechanical properties of the polyacrylamide substrates were determined by performing both unconfined and confined compression testing on cylindrical polyacrylamide specimens using a custom-built compression setup [28]. The typical sample dimensions were 8 mm in diameter and 4 mm in height. The displacements during each compression increment were controlled using a digital micrometer with a resolution of 1 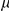
m. The resulting nominal force was measured using a 10 g load cell (A.L. Design). A total of 6–8 samples were tested in both confined and unconfined uniaxial compression.

For unconfined tests, gel samples were cast in a circular washer secured to the bottom of a 60 mm diameter plastic Petri dish. Following polymerization (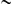
2–5 minutes), the washer was removed from the dish and the sample was hydrated and left covered at room temperature overnight to ensure hydrostatic (swelling) equilibrium. Prior to compression, the alignment of the setup with the sample was inspected to ensure pure compression along the nominal loading axis. The samples were compressed between the top platen of the compression setup and the bottom of the Petri dish with a nominal strain increment of 1–2%. Force values were obtained continuously during each 5-minute increment in order to detect any time-dependent relaxation of the material during the compression. The typical total applied compressive strain was 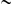
13–15%. After complete loading, the sample was successively unloaded using the same strain increments to record the entire loading-unloading cycle.

A Young's modulus of 9.64 kPa
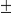
1.12 was calculated from the analysis of each stress-strain curve as 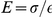, where 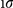 and 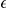 denote the nominal uniaxial engineering stress and strain.

In order to determine the Poisson's ratio for the polyacrylamide gels, cylindrical specimens were cast and polymerized in a confined Teflon sleeve (15 mm in diameter and about 8 mm in height). Samples were hydrated following the protocol described above. The samples were compressed following the same loading-unloading protocol used for the unconfined test. Using the determined Young's modulus value of the unconfined test case and observing that any further compression beyond an initial compression strain of 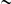
0.25% was not possible (due to the Poisson effect), Poisson's ratio was determined to be 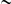
0.48–0.5 according to the following equation:
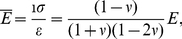
(1)where 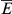 denotes the measured confined compression modulus, 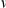 is the Poisson's ratio, and 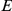 is the Young's modulus as determined from unconfined compression test. From this set of experiments, Poisson's ratio was taken to be 0.5, and the material behavior is described as linearly elastic, isotropic, and incompressible for all traction force calculations. The nominal volume fraction of fluorescent markers in the polyacrylamide substrates was 0.3%. The addition of the fluorescent microspheres had a negligible effect on the mechanical response of the substrate gels.

### Live cell imaging

**Figure 10:**
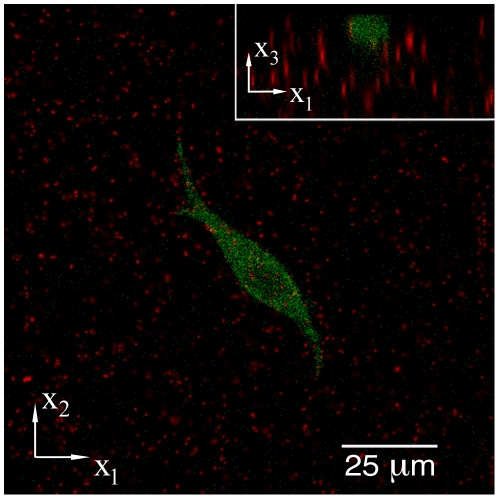
LSCM Cross-sectional Image. Representative LSCM image depicting the surface plane of a three-dimensional image stack, and a cross-sectional view of the scanned volume (inset). The fibroblast cell is showing GFP-actin in green, whereas the 0.5 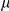
m fluorescent microspheres are shown in red.

Three-dimensional image stacks were acquired using a Nikon C-1 confocal system mounted on a TE-2000-U inverted optical microscope. A 40× CFI planar fluor air objective with a numerical aperture of 0.6 was used in all experiments. Three laser lines were used to image the cells and the fluorescent microparticles: An Argon (488 nm) laser for the GFP-actin, a green Helium Neon (543 nm) laser for the microparticles inside the polyacrylamide gels, and a red Helium Neon (633 nm) laser to excite Mitotracker Deep Red for mitochondrial labeling. Confocal stacks were acquired every 35 minutes for several hours at a resolution of 512×512×Z voxels (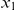
×
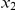
×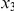), where Z ranges from 120 to 250 pixels. A three-dimensional pixel is commonly referred to as a voxel. Figure 10 shows a typical confocal image displaying GFP-actin (green) and 0.5 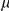
m fluorescent tracker particles (red). The inset in the upper right hand corner shows a cross-sectional (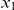
−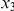) view through the thickness of the substrate. Typical imaging areas were between 150 to 200 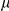
m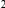 in-plane (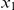, 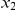) with an imaged volume depth of 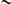
15–20 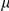
m. Images with a larger field of view were captured before and after experiments to ensure that measured displacements were not the result of contributions from neighboring cells. Typical volumetric image acquisition times were between 3 and 7 min depending on the final image size (e.g., the scan time for a 512×512×120 voxels image is 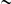
4 min). Physiological conditions were maintained by housing the entire confocal microscope inside a custom built temperature controlled chamber. The temperature was maintained at 37

C using a feedback controlled heater (Air-Therm ATX, World Precision Instruments), and cell media pH- and CO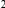 levels were maintained by the addition of arterial blood gas (5% CO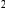, 20% O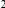, 75% N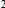) into the chamber.

### Calculation of displacements and traction forces

All displacement and strain fields were determined from LSCM volume stacks using a previously published technique [28]. In brief, using the recorded LSCM volume images, digital volume correlation (DVC) was used to achieve three-dimensional full field deformation measurements as an extension of the vision-based surface deformation measurement technique, known as digital image correlation (DIC). The main principle of DVC is based on the cross-correlation of individual cubic subsets that comprise each image per given time or deformation increment. The intensity distribution of the fluorescent tracker particles inside each subset is represented by a function pair 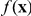 and 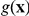 where 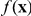 refers to the reference configuration and 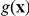 to the deformed or time incremented state. The displacement vector 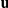 can then be calculated by determining the spatial location of the maximum peak of the correlation function
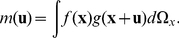
(2)


The cross-correlation function can be written using Fourier transforms as,
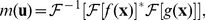
(3)where the Fourier transform of 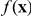 is defined as,
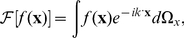
(4)and * denotes the complex conjugate. The discrete cross-correlation function can be computed efficiently by using the Fast Fourier Transform (FFT) algorithm. Determining the displacement vector 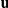 within sub-pixel accuracy generally requires fitting and interpolation of the correlation function near the peak. Various fitting models have been used in the past [39], [40], and a 3D quadratic polynomial fitting was previously demonstrated to accurately fit the correlation function near the peak thus providing improved sub-pixel accuracy.

Correlation-based techniques are generally advantageous over single particle tracking schemes since they rely on the correlation of image subsets with 40–80 particles on average and hence are much less sensitive to spatial and temporal intensity variation of individual particles. These algorithms such as DVC can also be run fully automated without the need of image thresholding to extract the full-field displacement fields [28], [41].

In order to establish the resolution sensitivity of this technique, fibronectin-functionalized polyacrylamide samples were prepared without cells and imaged under conditions identical to those used for live cell measurements. The three-dimensional displacement and strain fields were computed from these zero load images. Following statistical error analysis, these measurements indicated that the LSCM-DVC technique is capable of detecting displacement changes greater than 0.12 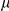
m under the above described imaging conditions, representing subpixel or submicron accuracy. The typical grid spacing of the digital volume correlation calculations was 2 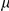
m but can be decreased at an increase in computational cost. The typical subset size was 64×64×64 voxels, which was shown to produce optimal correlation results for non-uniform displacement fields over a wide variety of particle volume fractions [28], [41]–[43]. All of the calculated and presented displacements, traction forces and surface normals are functions of the generalized Cartesian coordinates 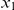, 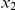, 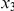 (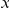, 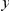, 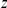).

#### Definition of the three-dimensional displacement vector

The three-dimensional displacement vector **u** is defined as follows,
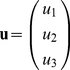
(5)with its magnitude given by
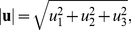
(6)where u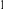, u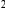, u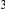 are the components of the displacement vector.

#### Traction calculations

At each time point the cell-induced strain fields were computed using a displacement-gradient technique [44]. Although the displacement field can be directly differentiated to obtain the material strains, the displacement-gradient technique provides a more robust solution that is less sensitive to noise in the displacement data, and hence provides improved accuracy. In brief, the local displacement field around each computed DVC grid point is approximated by
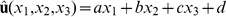
(7)where 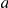,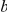,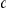, and 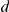 are constants to be determined by minimizing the following vector 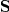 in the least-square sense using the measured displacement vector 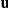


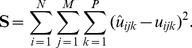
(8)


Point-wise least-squares minimzation of Eqs. 7 and 8 using a 3×3×3 pixel stencil or kernel yields the constants 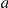,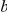,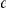 and 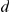 from which the full field strain tensor is constructed. A more detailed description of the displacement-gradient technique can be found in [44]. All calculated strains were found to be within the linear range of material behavior (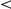
5%) and are expressed as the strain tensor denoted by 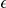.

In order to calculate traction stresses including surface tractions, the stress tensor 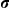 needs to be determined first, and is calculated based on the above experimentally determined constitutive properties (linearly elastic and incompressible [45]) as
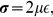
(9)where 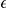 is the strain tensor and 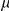 is the shear modulus, which can be related to Young's modulus 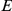 and Poisson's ratio 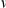 by
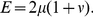
(10)The stress (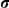) and strain (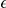) tensors can be written in matrix from,
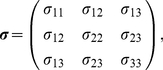
(11)and
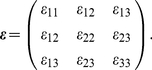
(12)Then, calculation of the traction forces involves using the well-known Cauchy relation [45],
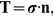
(13)where **T** is defined as the three-dimensional traction force vector, and **n** (components, 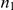, 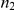, 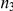) is the surface normal of an arbitrary plane on which **T** (components, 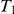, 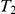, 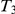) acts given by
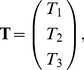
(14)and
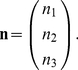
(15)The magnitude of the three-dimensional traction force vector is then defined as
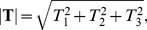
(16)All traction forces are presented as traction force per unit area in units of 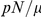
m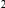 or Pascal (Pa). In the present study the 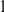 (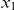) and 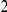 (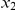) components refer to the in-plane quantities, and the 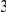 (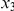) component corresponds to the out-of-plane or normal quantities.

#### Resolution and Measurement Sensitivity

Since the determination of the traction forces involves calculations of strains and experimental determination of material constants, the sensitivity of the LSCM-DVC technique in terms of traction forces needed to be assessed. This was accomplished by performing experiments using the same materials setup as for the measurements on migrating fibroblasts but without cells present. Hence, the measured displacements and calculated traction forces were solely due to thermal fluctuations and instrumental error, and thus established the sensitivity of the traction force calculations. Using standard statistical error analysis, the technique can accurately detect displacements and strains up to 0.12 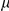
m and 0.5%, respectively, and hence by means of equations 9–13, stresses and traction forces that are greater than 80 Pa or 80 pN/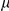
m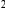 for all samples with a Young's modulus of 9.64 kPa [46]. As Figs. 2, 3, 4, 5, 6, 7 show, cellular traction magnitudes are 10–40 fold higher than the resolution limit of 80 Pa. A similar analysis of the maximum resolution for 2D TFM has been provided in the literature [34].

#### Global Force and Moment Balance

At each time point the sum of all forces and moments acting on any given control volume inside the polyacrylamide substrate was computed and static force and moment equilibrium were found to be satisfied. This was verified by comparing the net forces and net moments for a variety of different sized control volumes (N = 25) from several experiments on polyacrylamide gels with cells present and cells absent (control). For both systems the summation of forces and moments of each control volume was on the same order of magnitude, 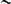
10
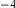
–10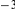 mN, and 10
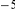
–10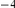 mN

mm for the net forces and net moments, respectively. These numbers represent the upper limit of net forces and net moments by choosing the entire three-dimensional image as a control volume. A simple force to strain analysis based on the above force magnitudes, the control volume area (i.e., entire imaging area) and the Young's modulus of the polyacrylamide substrates shows that the average zero-net strain value is 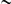
0.5%, which is consistent with the previously determined measurement strain and displacement sensitivities [46]. The results suggest that inertial effects do not play a significant role in the analysis and interpretation of the presented data, which is also reflected by the relatively low migration speeds of the fibroblasts used in this study.

## Supporting Information

Figure S1 **Close-up Cell Traction Image.** Expanded contour plot of the magnitude of the three-dimensional traction vector as shown in Figure 3(a). The dotted white line depicts the location and orientation of the plotted traction line profiles as seen in Figure 3(b).
(TIFF)

Figure S2 **Close-up Cell Traction Image.** Expanded cross-section depth contour plot of the magnitude of the three-dimensional traction vector as shown in Figure 3(c). The dotted green line depicts the location and orientation of the plotted traction line profiles as seen in Figure 3(d).
(TIFF)

Figure S3 **Close-up Cross-sectional Traction Image underneath Cell Nucleus.** Expanded view of Figure 6(b) showing the distribution of the magnitude of the three-dimensional traction vector (color contours) and the in-plane traction components (black arrows) underneath the cell nucleus as depicted in Figure 6(b).
(TIFF)
