# Supplementary figures and images for: Three-Dimensional Traction Force Microscopy: A New Tool for Quantifying Cell-Matrix Interactions
Source: PLoS One. 2011 Mar 29;6(3):e17833. doi: 10.1371/journal.pone.0017833 (PMC3066163; doi:10.1371/journal.pone.0017833)

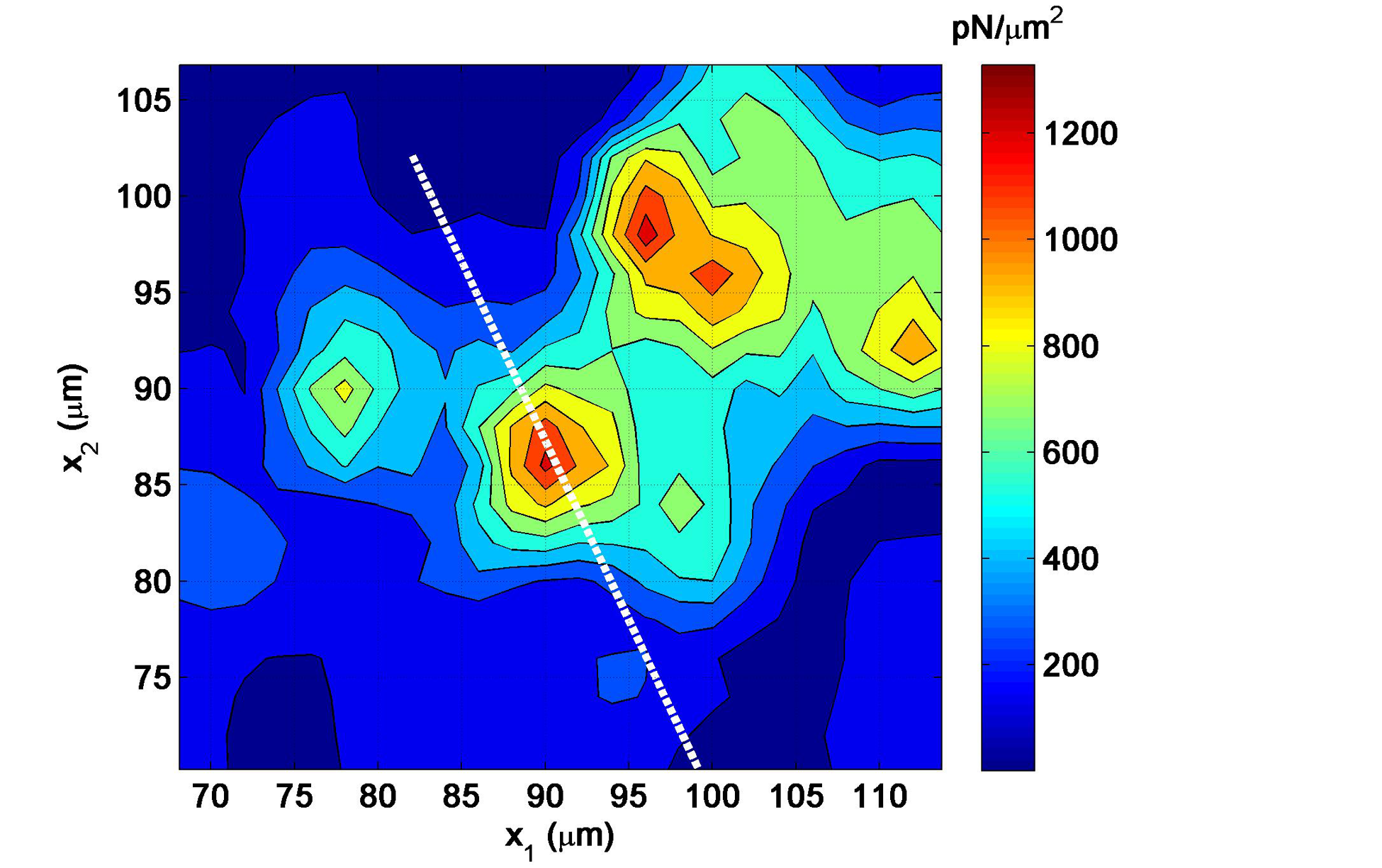

Supplement: Figure S1 — Close-up Cell Traction Image. Expanded contour plot of the magnitude of the three-dimensional traction vector as shown in Figure 3(a). The dotted white line depicts the location and orientation of the plotted traction line profiles as seen in Figure 3(b). (TIFF) [file pone.0017833.s001.tif]

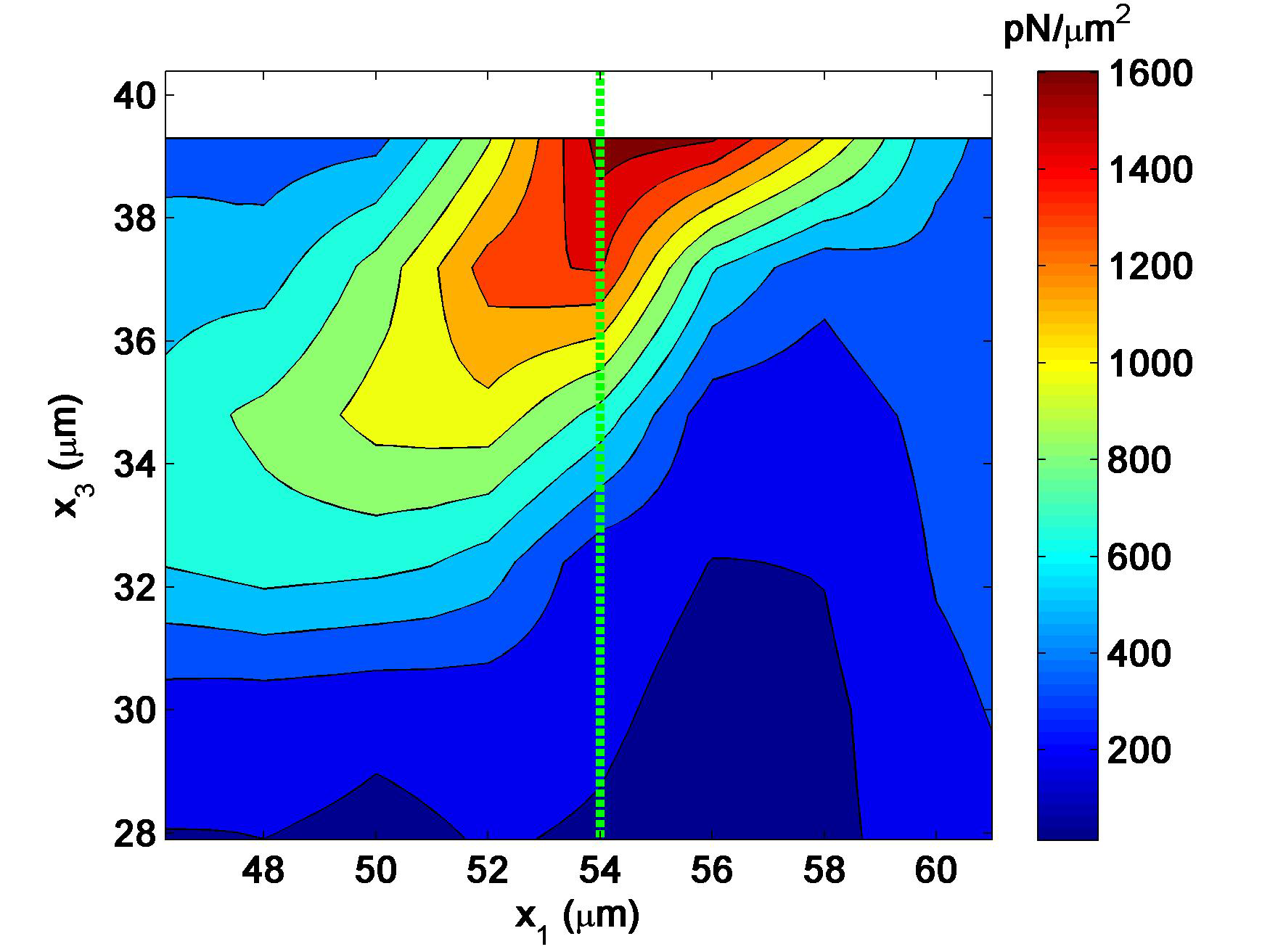

Supplement: Figure S2 — Close-up Cell Traction Image. Expanded cross-section depth contour plot of the magnitude of the three-dimensional traction vector as shown in Figure 3(c). The dotted green line depicts the location and orientation of the plotted traction line profiles as seen in Figure 3(d). (TIFF) [file pone.0017833.s002.tif]

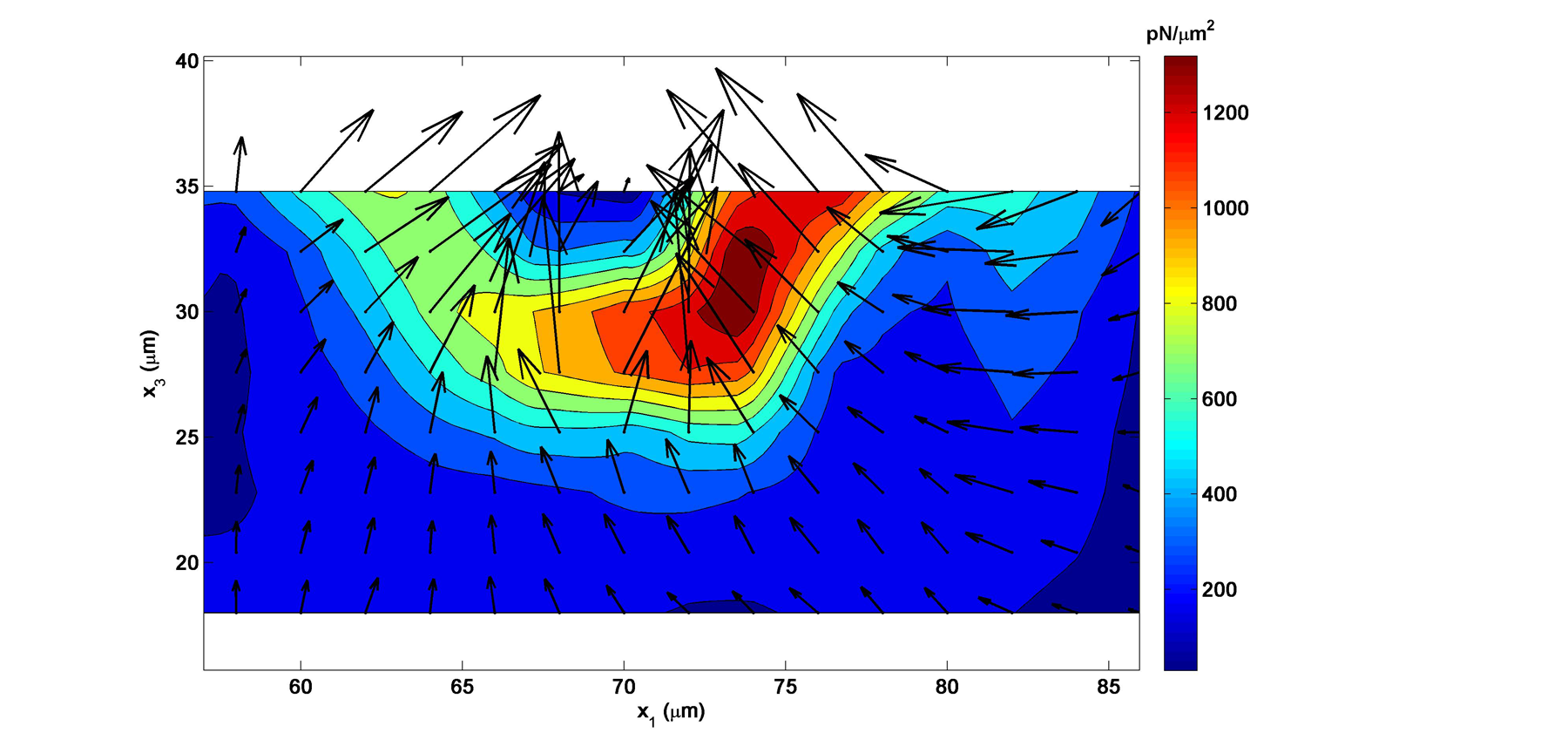

Supplement: Figure S3 — Close-up Cross-sectional Traction Image underneath Cell Nucleus. Expanded view of Figure 6(b) showing the distribution of the magnitude of the three-dimensional traction vector (color contours) and the in-plane traction components (black arrows) underneath the cell nucleus as depicted in Figure 6(b). (TIFF) [file pone.0017833.s003.tif]
